# A Fully Phase‐Modulated Metasurface as An Energy‐Controllable Circular Polarization Router

**DOI:** 10.1002/advs.202001437

**Published:** 2020-07-16

**Authors:** Yueyi Yuan, Shang Sun, Yang Chen, Kuang Zhang, Xumin Ding, Badreddine Ratni, Qun Wu, Shah Nawaz Burokur, Cheng‐Wei Qiu

**Affiliations:** ^1^ Department of Microwave Engineering Harbin Institute of Technology Harbin 150001 China; ^2^ Department of Electrical and Computer Engineering National University of Singapore Singapore 117583 Singapore; ^3^ Advanced Microscopy and Instrumentation Research Center Harbin Institute of Technology Harbin 150080 China; ^4^ LEME, UPL Univ Paris Nanterre Ville‐d'Avray F92410 France

**Keywords:** circular polarization, energy‐control, metasurfaces, phase modulation, routers

## Abstract

Geometric metasurfaces primarily follow the physical mechanism of Pancharatnam–Berry (PB) phases, empowering wavefront control of cross‐polarized reflective/transmissive light components. However, inherently accompanying the cross‐polarized components, the copolarized output components have not been attempted in parallel in existing works. Here, a general method is proposed to construct phase‐modulated metasurfaces for implementing functionalities separately in co‐ and cross‐polarized output fields under circularly polarized (CP) incidence, which is impossible to achieve with solely a geometric phase. By introducing a propagation phase as an additional degree of freedom, the electromagnetic (EM) energy carried by co‐ and cross‐polarized transmitted fields can be fully phase‐modulated with independent wavefronts. Under one CP incidence, a metasurface for separate functionalities with controllable energy repartition is verified by simulations and proof‐of‐principle microwave experiments. A variety of applications can be readily expected in spin‐selective optics, spin‐Hall metasurfaces, and multitasked metasurfaces operating in both reflective and transmissive modes.

## Graphical abstract

A general scheme based on a phase‐modulated metasurface for simultaneous and independent manipulation of co‐ and cross‐polarized wavefronts is proposed, which breaks through the inherent limitation of the geometric phase principle and achieves full energy modulation in output fields. The energy proportion between two orthogonal CP output functionalities can also be artificially manipulated as an extra freedom of degree for light manipulation.

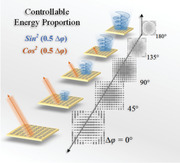

## 1 Introduction

Metasurfaces have developed into a proficient research area in recent years. By judiciously tailoring the transmission/reflection responses of sub‐wavelength meta‐atoms, metasurfaces have enabled various applications by modulating polarization, amplitude or phase of light.^[^ ^1^ ^]^ In optical and terahertz regimes, metasurfaces exhibit distinguished abilities for applications in achromatic focusing lenses,^[^ ^2^, ^3^, ^4^ ^]^ spin‐to‐orbital angular momentum conversion,^[^ ^5^, ^6^, ^7^, ^8^ ^]^ computed hologram imaging,^[^ ^9^, ^10^, ^11^, ^12^ ^]^ and flat wave plate.^[^ ^13^, ^14^, ^15^ ^]^ Moreover, metasurfaces also find diverse applications in radio and microwave frequencies, such as high‐gain antennas,^[^ ^16^ ^]^ cloaking,^[^ ^17^, ^18^ ^]^ holograms,^[^ ^19^ ^]^ biomedical diagnosis and treatment,^[^ ^20^, ^21^ ^]^ radar cross section (RCS) reduction,^[^ ^22^ ^]^ and vortex beam for wireless communication systems.^[^ ^23^, ^24^, ^25^ ^]^

As known, geometric metasurface is a convenient method for phase‐wavefront engineering.^[^ ^6^, ^7^, ^8^, ^10^, ^23^, ^26^, ^27^, ^28^, ^29^, ^30^, ^31^, ^32^, ^33^ ^]^ However following the physical mechanism of Pancharatnam–Berry (PB) phases,^[^ ^34^ ^]^ there are two major inherent restrictions. The first one is the conjugated symmetry, which would exhibit equal and opposite phase profiles under left‐handed circularly polarized (LHCP) and right‐handed circularly polarized (RHCP) illumination.^[^ ^6^, ^10^, ^26^ ^]^ For instance, a geometric phase based converging metalens for one circular polarization (CP) incidence state will act as a diverging one for the other orthogonal CP incidence.^[^ ^35^ ^]^ To overcome this limitation, propagation phase is applied to decouple the conjugate responses of geometric phase, and generate independent wavefronts in two cross‐polarized output fields with LHCP and RHCP incident illuminations.^[^ ^7^, ^36^, ^37^, ^38^, ^39^, ^40^, ^41^ ^]^ On the other hand, the generation of PB phase is accompanied with a space‐variant conversion of polarization states following the path on the Poincaré sphere,^[^ ^34^ ^]^ leading to the fact that PB phase based metasurfaces can only phase‐modulate cross‐polarized output field.^[^ ^27^, ^28^, ^29^, ^30^, ^31^, ^32^, ^33^, ^34^ ^]^ In existing works, great efforts have been devoted to enhance the cross‐polarized conversion efficiency, which indicates that geometric metasurfaces should infinitely approach the perfect half wave‐plate and require strictly *π*‐phase difference between linear orthogonal polarizations of birefringent elements.^[^ ^41^, ^42^ ^]^ Hence, when the *π*‐phase difference cannot be rigorously satisfied, there would always exist output energy carried by CP field polarization state as incidence, which cannot be phase‐modulated by solely geometric phase to achieve any specific functionality. Besides digging deeply in the cross‐polarized channel, it does make sense to exploit a general formalism to route output energy in both co‐ and cross‐polarized output CP channels.

**Figure 1:**
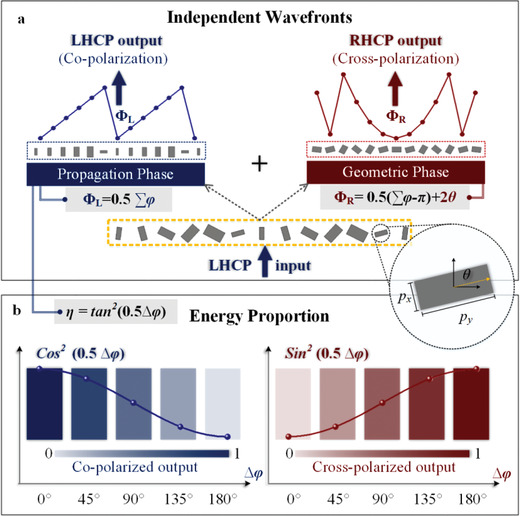
Schematic principle of proposed phase modulation strategy. a) For independent wavefront modulation, the phase scheme contains two parts: one is propagation phase for copolarized output, which is determined by the summation of phase delay along *x*‐ and *y*‐direction (∑*φ* = *φ_xx_* + *φ_yy_*), the other is geometric phase for cross‐polarized wavefront by rotating meta‐atoms (*φ*’ = 2*θ*). b) As for energy controllable circular polarization router, the energy ratio *η* between co‐ and cross‐polarized output waves can be tailored by propagation phase with the phase difference between *x*‐ and *y*‐direction (∆*φ* = *φ_xx_* – *φ_yy_*).

Herein, we propose a general scheme to simultaneously phase‐manipulate both copolarized and cross‐polarized components to achieve independent wavefronts and energy repartition, as schematically illustrated in **Figure** **1**. For output fields keeping the incidence polarization state, the wavefront can be modulated by propagation phase, the summation of phase delays along fast and slow axes would result in phase profile with no sensitivity to CP handedness, as shown in Figure 1a. Meanwhile for the cross‐polarized output, geometric phase is the main modulation factor to fulfill the preset functionality that is independent of copolarized wavefront. Bi‐functional metasurface is then constructed by using this approach, which is designed under LHCP illumination to generate copolarized converging beam and cross‐polarized vortex beam, validating the availability and independence of two output channels. Moreover, the energy distributed in two orthogonal output CP channels can also be tailored by propagation phase, and the evolution of energy ratio between two polarizations is demonstrated with the phase difference between fast and slow axes (∆*φ*), as shown in Figure 1b. Proof‐of‐concept experiments are conducted in microwave region to validate the theoretical paradigm. Metasurfaces based on the generalized formulism could make full utilization of EM energy carried by both co‐ and cross‐polarized output fields, which would be no longer limited by the cross‐polarization conversion efficiency, and could be further extended to other frequency regimes.

## 2 Phase Modulation Scheme and Meta‐Atom Design

### 2.1 General Scheme for Fully Phase‐Modulated Metasurface

Let us start with the derivation of the proposed mechanism. It is supposed that when input with LHCP state ${\vec{\varsigma }}^{+}=\left[\begin{array}{c} \varsigma_{1}^{+}\\ \varsigma_{2}^{+} \end{array}\right]=\left[\begin{array}{c} 1\\ i \end{array}\right]$ passes through a lossless, passive, reciprocal and matched ideal metasurface model, the output EM fields would inevitably possess two components, one keeping similar polarization state as the incidence ${\vec{\gamma }}^{+}$ and the other owning the orthogonally converted polarization${\left({\vec{\gamma }}^{+}\right)}^{*}={\vec{\gamma }}^{-}$, where ^*^ denotes the complex conjugate for reversed CP state from the incidence. The transformation process is consistently implemented through the entire metasurface extent, which can be described as ${\vec{\varsigma }}^{+}\to {\vec{\gamma }}^{+}+{\vec{\gamma }}^{-}$. It is desired that the independent spatial functional wavefronts can be imposed into each output CP components, which should be achieved by the Jones matrix *J*(*x*, *y*) of each element with location (*x*, *y*)

(1) $$J\left(x,y\right)\cdot {\vec{\varsigma }}^{+}=e^{i\Phi_{1}^{+}\left(x,y\right)}\cdot {\vec{\gamma }}^{+}+e^{i\Phi_{2}^{+}\left(x,y\right)}\cdot {\left({\vec{\gamma }}^{+}\right)}^{*}$$

**Figure 2:**
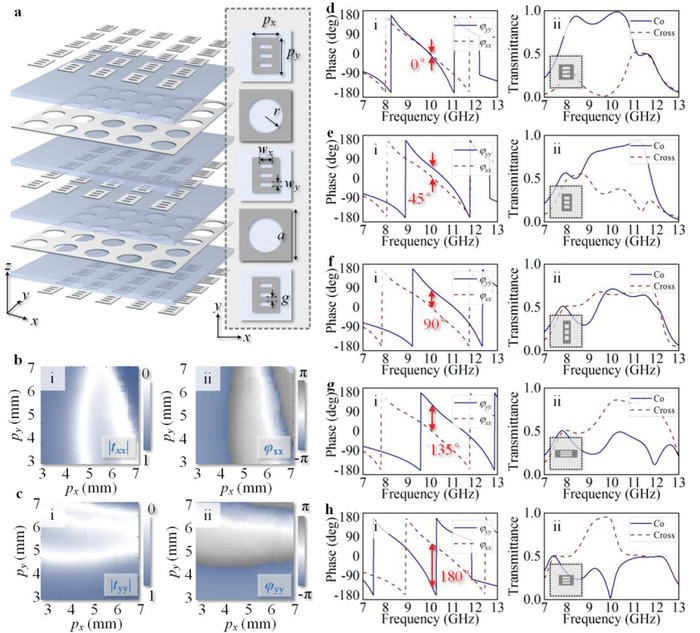
Simulation verification of the proposed meta‐atom. a) The topological structure of an array of meta‐atoms, and the inset shows the specific layouts of each layer of meta‐atom. b,c) Simulated transmission coefficients mappings under *x*‐linearly polarized (b) and *y*‐linearly polarized (c) incidences against variable dimension sizes of patch layer of meta‐atom, where (i) shows the amplitude profiles and (ii) is the phase profiles. d–h) The simulated transmission coefficient profiles of five representative meta‐atoms with distinct sizes of rectangular patch layer for ∆*φ* = 0°, 45°, 90°, 135°, and 180°, respectively, where (i) is the phase responses under linearly polarized incident waves and (ii) is the corresponding co‐ and cross‐polarized amplitude responses under LHCP incidence.

In order to implement the scheme expressed in Equation (1), we apply birefringent wave‐plate as phase‐shifting block units to achieve the required Jones matrix,^[^ ^43^, ^44^ ^]^ The transmission response of basic meta‐atom is considered as $T=\left[\begin{array}{cc} |t_{xx}|\cdot e^{i\varphi_{xx}} & 0\\ 0 & |t_{yy}|\cdot e^{i\varphi_{yy}} \end{array}\right]$, where |*t_xx_*|, |*t_yy_*|, *φ_xx_*, and *φ_yy_* are the corresponding linear amplitudes and phase delays along *x*‐ and *y*‐axis. Here, the linear amplitudes satisfy |*t_xx_*| = |*t_yy_*| = 1 and the phase difference between two orthogonal linear polarizations is defined as ∆*φ* = *φ_xx_* – *φ_yy_*. With the characteristics of wave‐plate model element, Equation (1) can further be transformed into (the derivation process is detailed the Supporting Information)

(2) $$\begin{array}{ccc} J\left(\theta \right)\cdot {\vec{\varsigma }}^{+} & = & \cos\left(\frac{1}{2}\Delta \varphi \right)\cdot e^{i\cdot \frac{1}{2}\sum \varphi }\cdot {\vec{\varsigma }}^{+}\\ & & +\;\sin\left(\frac{1}{2}\Delta \varphi \right)\cdot e^{i\cdot \frac{1}{2}\left(\sum \varphi -\pi \right)}\cdot e^{i\cdot 2\theta }\cdot {\vec{\varsigma }}^{-} \end{array}$$

where *θ* is the rotation angle of the meta‐atom, originating from the rotation matrix $M\left(\theta \right)=\left[\begin{array}{cc} \cos\theta & \sin\theta \\ -\sin\theta & \cos\theta \end{array}\right]$, and the summation of propagation phase along fast and slow axis is expressed as ∑*φ* = *φ*_*xx*_ + *φ*_*yy*_. It can be seen in Equation (2) that there are theoretically two CP output components under one CP incidence; the copolarized one owning the same CP state as the input ${\vec{\varsigma }}^{+}$, and the other with the opposite polarization state ${\vec{\varsigma }}^{-}$ representing the cross‐polarized output. The phase profile imprinted to the copolarized component $\Phi_{1}^{+}=\frac{1}{2}\sum \varphi$, implies that wavefront of the copolarized output can be directly phase‐tuned by the summation of propagation phase along fast and slow axes, which is further tailored by dimensions of utilized birefringent meta‐atom. Meanwhile, phase profiles introduced in the cross‐polarized field, which can be considered as geometric phase assisted by propagation phase, is described as $\Phi_{2}^{+}=\frac{1}{2}\left(\sum \varphi -\pi \right)+2\theta$, indicate that the required cross‐polarized function can be realized independently through additional rotation of the meta‐atom. That is to say, the geometric phase provided by rotating angle would be $\theta =\frac{1}{2}\left(\Phi_{2}^{+}-\Phi_{1}^{+}+\frac{\pi }{2}\right)$. This phase‐tuning process is schematically illustrated in Figure 1a. Furthermore, the amplitude of co‐ and cross‐polarized parts contain a cos‐ and sin‐form coefficients relative to the difference of propagation phase along fast and slow axes ∆*φ*, which can be regarded as a weight factor for tuning the energy proportion carried by co‐ and cross‐polarized output fields. The energy ratio is expressed by $\eta =\tan^{2}\left(\frac{\Delta \varphi }{2}\right)$, and the manipulation schematic is shown in **Figure** **2**b. Since propagation phase along fast and slow axes are independent, the phase‐modulated wavefronts and energy weight factor *η* can be adjusted separately. It is worth noting that conventional geometric metasurfaces elaborately manufactured aiming at high cross‐polarization conversion efficiency (with ∆*φ* → *π*), can be regarded as a special case of the generalized formalism, and the strict condition of high cross‐polarization conversion efficiency is released here.

### 2.2 Meta‐Atom Design

Here we apply a passive and reciprocal meta‐atom with dual‐fold symmetric structure as the basic element for the metasurface construction, which is schematically shown in Figure 2a and specifically descripted in the Supporting Information. This meta‐atom is composed of five metallic layers and four dielectric layers with same thickness *h* = 1 mm. The constant parameters are the period *a* = 8.8 mm and radius of circle aperture in even metallic layers *r* = 3.0 mm, the odd metallic patch layer is the crucial part for phase‐tuning the co‐ and cross‐polarized output components. Figure 2b,c exhibits the transmission coefficients of meta‐atoms under linearly polarized incidence with variable dimension sizes (width *p_x_* and length *p_y_*) of rectangular patch in odd metallic layers. It is illustrated that under linearly polarized incidence, the phase combination ∑*φ* can fully cover the range of 2*π* by tailoring the sizes of the metallic patch, guaranteeing that the versatile wavefronts can be performed by discretized propagation phase distribution. Five representative meta‐atoms with distinct *p_x_* and *p_y_* are selected to verify the phase‐tuned co‐ and cross‐polarized transmission coefficients as shown in Figure 2d–h, where i) presents the phase profiles *φ_xx_* and *φ_yy_* under linearly polarized incidences and ii) is the corresponding co‐ and cross‐polarized amplitudes under LHCP incidence. It can be seen that the two CP amplitude values at working frequency of 10 GHz exhibit different tendency with the increase of phase difference between the orthogonal linear polarizations ∆*φ*, which is in agreement with the derivation of Equation (2). Such characteristics pave the basic step to achieve the further manipulation of orthogonal output CP functionalities with controllable energy distribution.

## 3 Demonstration of the Energy‐Controllable Phase‐Modulated Metasurface

**Figure 3:**
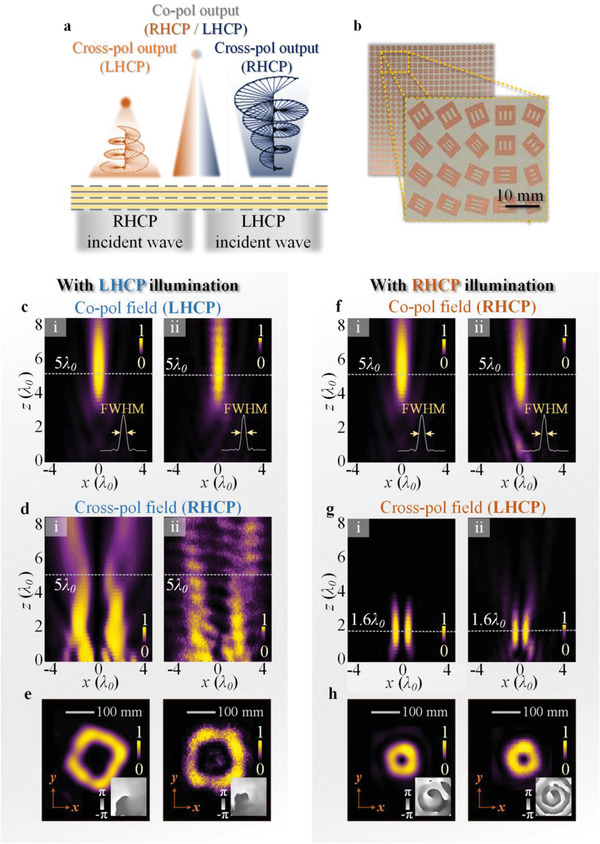
Demonstration of proposed metasurfaces with tri‐functionalities under orthogonal CP incidences. a) Schematic for generating converging beam, vortex beam carrying OAM mode with *l* = 1 and focused OAM with *l* = −1 under LHCP and RHCP illuminations. b) Photography of fabricated sample. c,d) Under LHCP incidence, the energy intensity of focusing beam in copolarized field in the *xoz* plane (c) and OAM beam in cross‐polarized field in the *xoz* plane (d), e) energy intensities of vortex beam carried by cross‐polarized fields in the *xoy* plane at *z* = 5*λ*_0_, the insets show the corresponding and phase distributions. f‐h) When flipping the incident state to RHCP incidence, the energy distributions of the copolarized focusing beam (f) and the cross‐polarized converging OAM beam (g) in the *xoz* plane, and the cross‐polarized energy intensities in the *xoy* plane at *z* ≈ 1.6*λ*_0_ (h), the insets show the corresponding and phase distributions. (i,ii) exhibit simulation and measurement results at 10 GHz respectively.

To demonstrate the general method established above, a metasurface is designed operating under LHCP input to obtain separate wavefronts of copolarized converging beam and cross‐polarized vortex beam, as schematically shown in **Figure** **3**a and labelled as metasurface‐1. The spatial phase distributions of these two beams are described as follows

(3a) $$\Phi_{1}^{+}\left(x,y\right)=\Phi_{conv}\left(x,y\right)=\frac{2\pi }{\lambda_{0}}\left(\sqrt{{f_{1}}^{2}+\left(x^{2}+y^{2}\right)}-\left|f_{1}\right|\right)$$

(3b) $$\begin{array}{cc} \Phi_{2}^{+}\left(x,y\right) & =\Phi_{1}^{+}\left(x,y\right)+2\theta \\ & =\Phi_{OAM}\left(x,y\right)=l\cdot \arctan\left(y/x\right) \end{array}$$

where the focal length *f*_1_ = 5*λ*_0_, topological charge of *l* = 1 and the working wavelength *λ*_0_ = 30 mm. The fabricated metasurface‐1 sample is exhibited in Figure 3b. Figure 3c–h illustrates the results of the metasurface‐1 performed in microwave region at 10 GHz, where (i,ii) present the simulation and measurements, respectively. Under LHCP wave illumination, the energy intensity of the copolarized output along propagation direction in the *xoz* plane is displayed in Figure 3c, showing that the copolarized energy is successfully focused into the preset plane *z* = 5*λ*_0_. The full width at half maximum (FWHM) of normalized intensity profiles of the focal spot, shown in the inset of Figure 3c, is 0.89*λ*_0_ in simulation and 0.87*λ*_0_ in measurement, indicating that a focus spot with high quality can be produced. Under the same illumination, the vortex beam generated in cross‐polarized output field is displayed in Figure 3d,e. The normalized energy intensities in the *xoz* plane succeed to perform the intrinsic hollow distribution along propagation direction, and the doughnut‐shape intensity and helical phase pattern in the *xoy* plane (at *z* = 5*λ*_0_) further represent the generation of orbital angular momentum (OAM) mode with *l* = 1 in the transmitted cross‐polarized field. The above results illustrate that the independent converging beam and vortex beam are separately produced in co‐ and cross‐polarized channels under the LHCP incidence.

When the handedness of incident beam is changed to RHCP, the phase profiles of co‐ and cross‐polarized output wavefronts would change to

(4a) $$\Phi_{1}^{-}\left(x,y\right)=\Phi_{conv}\left(x,y\right)=\frac{2\pi }{\lambda_{0}}\left(\sqrt{{f_{1}}^{2}+\left(x^{2}+y^{2}\right)}-\left|f_{1}\right|\right)$$

(4b) $$\begin{array}{cc} \Phi_{2}^{-}\left(x,y\right) & =\Phi_{1}^{-}\left(x,y\right)-2\theta \\ & =\frac{2\pi }{\lambda_{0}}\left(\sqrt{{f_{2}}^{2}+\left(x^{2}+y^{2}\right)}-\left|f_{2}\right|\right)-l\cdot \arctan\left(y/x\right) \end{array}$$

It can be seen from Equation (4), the wavefront provided by propagation phase is kept unchanged ($\Phi_{1}^{-}\left(x,y\right)=\Phi_{1}^{+}\left(x,y\right)$) and the copolarized output wavefront is still as a converging beam with focal length 5*λ*_0_ as shown in Figure 3f. The simulated and measured FWHM of normalized intensity of focal spot (inset of Figure 3f) are both near to 0.9*λ*_0_, which is identical to that of copolarized output with LHCP incidence. Meanwhile, the cross‐polarized wavefront could exhibit an extra 3rd functionality, which can be regarded as the linear combination of focusing beam and vortex beam, instead of simply conjugated function as metasurface based on solely geometric phase exhibits. Accordingly, the phase profile can be described by Equation (4b). The focal length of converging vortex beam carrying OAM mode can be approximately calculated to be $f_{2}=2f_{1}-\frac{3(x^{2}+y^{2})}{4(\sqrt{x^{2}+y^{2}+{f_{1}}^{2}}-\left|f_{1}\right|)}\approx 1.6\lambda_{0}$. Thus, the cross‐polarized wavefront performs a converging vortex beam with OAM mode *l* = −1. The simulated (i) and measured (ii) energy intensities in the *xoz* plane displayed in Figure 3g, showing that the focused hollow energy distribution exhibits the focal length with 1.6*λ*_0_, agreeing with the theoretical preset. The corresponding compact energy ring and helical phase pattern with *l* = −1 in the *xoy* focal plane (*z* = 1.6*λ*_0_) are shown in Figure 3h, which demonstrates the focusing vortex beam generation. This formalism can be extended to other frequency band, and we further demonstrate the feasibility in optical regime with working wavelength *λ*_0(opt)_ = 1550 nm (detailed in the Supporting Information). Moreover, the scheme for fully phase‐modulation can also be applied in reflection‐mode metasurfaces, as long as the meta‐atom system can independently impose propagation and geometric phase responses.^[^ ^37^ ^]^

**Figure 4:**
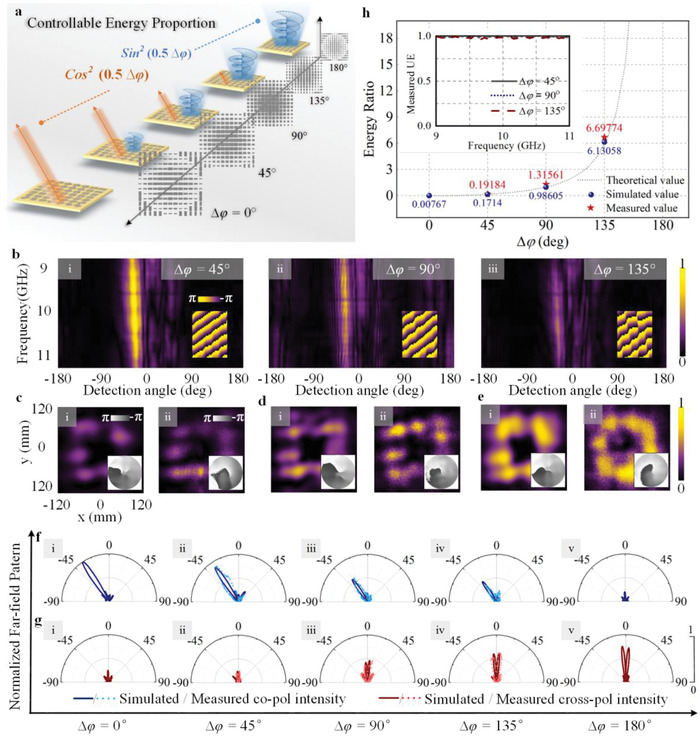
a) The schematic of the evolution of energy distributed in co‐ and cross‐polarized output fields for metasurface‐2. b) Measured copolarized far‐field intensity against detection angle and frequency, where (i–iii) represent for ∆*φ* = 45°, 90°, and 135°, the insets show the corresponding simulated deflected phase‐fronts in the *xoz* plane at 10 GHz. c‐e) Energy intensity of cross‐polarized vortex beam in the *xoy* plane at *z* = 5*λ*_0_ with ∆*φ* = 45° (c), 90° (d) and 135° (e); the insets represent corresponding phase distributions. f,g) Normalized far‐field patterns for refraction (copolarized output) (f) and vortex beam carrying OAM mode with *l* = 1 (cross‐polarized output) (g) which are all normalized to the maximum intensity in copolarized pattern with ∆*φ* = 0°. h) Theoretical, simulated and measured energy ratio with the variation of ∆*φ*, the inset shows the measured utilization efficiency of metasurfaces‐2.

As presented in our proposed formalism, the proportion of energy distributed in the two output orthogonal CP channels can be flexibly manipulated through tailoring the phase difference between linear birefringent polarizations (denoted as ∆*φ*). Here, the evolution of energy distribution in the two output CP channels with ∆*φ* is demonstrated by metasurface‐2 as schematically shown in **Figure** **4**a, which is designed for an operation under LHCP illumination to generate a deflected beam at −35° ($\Phi_{1}^{+}\left(x,y\right)=\Phi_{\mathrm{refra}}\left(x,y\right)=\frac{2\pi }{\lambda_{0}}\sin\alpha \cdot x$) in copolarized channel and vortex beam carrying OAM mode with topological charge *l* = 1 (same as in Equation (3b)) in cross‐polarized channel. It is important to note that, the energy ratio between co‐ and cross‐polarized channels can be maintained when the input CP state is flipped, since it is only related to propagation phase. To demonstrate the independent control of energy ratio *η*, a series of metasurfaces‐2 working under the LHCP wave illumination, are proposed to achieve the same functionalities with variable ∆*φ* from 0 to *π* with an interval of *π*/4, respectively. Metasurfaces‐2 with ∆*φ* = 45°, 90°, and 135° are fabricated to validate the controllable energy repartition, and measured results are shown in Figure 4b–e. It can be seen that copolarized far‐field intensities within frequency band 9–11 GHz of metasurfaces‐2 (with ∆*φ* = 45° (i), 90° (ii), and 135° (iii)) is getting weaker with the increase of ∆*φ* as shown from Figure 4b‐i to iii. Meanwhile, the cross‐polarized energy intensity of vortex beam generated by corresponding metasurfaces‐2 changes into stronger intensity, which can be compared in Figure 4c–e, where (i) is for simulation and (ii) is for measurement. The evolution of co‐ and cross‐polarized far‐field patterns against ∆*φ* are exhibited in Figure 4f,g, where the far‐field output wavefront is a refracted beam at −35° and vortex beam, respectively. It can be observed that with the increase of ∆*φ*, the far‐field intensities of copolarized refraction and cross‐polarized vortex beam exhibit opposite tendencies, which is in agreement with the energy coefficient cos^2^(0.5∆*φ*) and sin^2^(0.5∆*φ*) in Equation (2) (square of amplitude coefficient). Then, we also calculate the energy ratio *η* = tan^2^(0.5∆*φ*) according to Equation (2), which is illustrated by the gray dotted line in Figure 4h. The simulated energy ratios of metasurface‐2 with different ∆*φ* are represented by blue spheres (the value with ∆*φ* = 180° is about 152.7, which is not shown here), and the three measurement results are presented by red stars. The agreement between theory, simulation and measurement indicates the feasibility of proposed scheme. Furthermore, it is important to evaluate the efficiency of the proposed metasurfaces (defined in the Supporting Information). Inset of Figure 4h displays measured utilization efficiencies (UEs) of proposed metasurface‐2s are approaching 1, which is calculated as the ratio between summation of phase‐modulated co‐ and cross‐polarized output and the total transmitted output, meaning that almost all the transmitted energy can be employed to achieve the preset functionalities. The extra degree of freedom for distributable energy in the two orthogonal output CP channels does not result in the loss of transmitted energy, which is further analyzed in the Supporting Information.

## 4 Conclusion

To summarize, we have presented a general strategy to simultaneously and independently manipulate both co‐ and cross‐polarized transmitted outputs under CP incidence. Metasurfaces are proposed to validate the scheme to achieve full usage of energy in transmission, which verifies that our scheme can be a good alternative to address the inherent limitation of PB phase based metasurfaces that only operate in cross‐polarization manner. Under one particular CP input, the energy distributed in two output orthogonal channels can be artificially assigned, providing an extra degree of freedom for multifunctionality control. Simulations and measurements have been performed on the proposed structure and the results have validated the predetermined wavefronts. Nearly 100% utilization of transmitted fields can be exploited in both CP channels leaving no polarization behind. The paradigm can be extended to reflection‐type metasurfaces working in both optical and microwave regions.

## Conflict of Interest

The authors declare no conflict of interest.

## Supporting information

Supporting Information
