# Supplementary material for: A Fully Phase‐Modulated Metasurface as An Energy‐Controllable Circular Polarization Router
Source: Adv Sci (Weinh). 2020 Jul 16;7(18):2001437. doi: 10.1002/advs.202001437 (PMC7509705; doi:10.1002/advs.202001437)
Supplement: Supplementary file 1 — Supporting Information [file ADVS-7-2001437-s001.pdf]

Copyright WILEY-VCH Verlag GmbH & Co. KGaA, 69469 Weinheim, Germany, 2020.

Supporting Information

A Fully Phase-Modulated Metasurface as An Energy-Controllable Circular Polarization Router

Yueyi Yuan, Shang Sun, Yang Chen, Kuang Zhang, Xumin Ding*, Badreddine Ratni, Qun Wu, Shah Nawaz Burokur*, and Cheng-Wei Qiu**

Text S1. Derivation process of Jones matrix for meta-atom

Text S2. Design principle of meta-atom

Text S3. Fabrication process of microwave metadevice sample

Text S4. Measurement system in microwave region

Text S5. Scheme demonstration in optical regime

Text S6. Evaluation of efficiencies of proposed metadevices

Text S1. Derivation process of Jones matrix for meta-atom

In this part, the specific derivation process of Jones matrix is exhibited. Based on linear polarization basis, the Jones matrix with inherent linear responses along *x*- and *y*-directions and rotation characteristics is given as:

$$\mathbf{J}(\theta)=\mathbf{M}(\theta)^{\mathrm{T}}\times\mathbf{T}(0)\times\mathbf{M}(\theta)=\mathbf{M}(\theta)^{\mathrm{T}}\begin{bmatrix}\mathbf{t_{xx}} & 0\\ 0 & \mathbf{t_{yy}}\end{bmatrix}\mathbf{M}(\theta) \tag{S1}$$

where $\mathbf{M}(\theta)=\begin{bmatrix}\cos\theta & \sin\theta\\ -\sin\theta & \cos\theta\end{bmatrix}$ is the rotation matrix and θ is the rotation angle, $\mathbf{T}(0)$ is the linear transmission coefficient of a symmetric meta-structure, and $\mathbf{t_{xx}}=|t_{xx}|e^{i\varphi_{xx}}$, $\mathbf{t_{yy}}=|t_{yy}|e^{i\varphi_{yy}}$ are the linear transmission coefficients imposed by meta-atoms with $\theta = 0$, $|t_{xx}|$, $|t_{yy}|$, φ_{xx} and φ_{yy} are the corresponding amplitudes and phase delays. With the expansion calculation, the Jones matrix is calculated as:

$$\begin{aligned}\mathbf{J}(\theta)&=\begin{bmatrix}\cos\theta & -\sin\theta\\ \sin\theta & \cos\theta\end{bmatrix}\begin{bmatrix}|t_{xx}|e^{i\varphi_{xx}} & 0\\ 0 & |t_{yy}|e^{i\varphi_{yy}}\end{bmatrix}\begin{bmatrix}\cos\theta & \sin\theta\\ -\sin\theta & \cos\theta\end{bmatrix}\\
&=\begin{bmatrix}\cos^2\theta\cdot|t_{xx}|e^{i\varphi_{xx}}+\sin^2\theta\cdot|t_{yy}|e^{i\varphi_{yy}} & \sin\theta\cos\theta\cdot|t_{xx}|e^{i\varphi_{xx}}-\sin\theta\cos\theta\cdot|t_{yy}|e^{i\varphi_{yy}}\\ \sin\theta\cos\theta\cdot|t_{xx}|e^{i\varphi_{xx}}-\sin\theta\cos\theta\cdot|t_{yy}|e^{i\varphi_{yy}} & \sin^2\theta\cdot|t_{xx}|e^{i\varphi_{xx}}+\cos^2\theta\cdot|t_{yy}|e^{i\varphi_{yy}}\end{bmatrix}\\
&=\frac{1}{2}(|t_{xx}|e^{i\varphi_{xx}}-|t_{yy}|e^{i\varphi_{yy}})\cdot\begin{bmatrix}\cos2\theta & \sin2\theta\\ \sin2\theta & -\cos2\theta\end{bmatrix}+\frac{1}{2}(|t_{xx}|e^{i\varphi_{xx}}+|t_{yy}|e^{i\varphi_{yy}})\cdot\begin{bmatrix}1 & 0\\ 0 & 1\end{bmatrix}\\
&=J_1+J_2\end{aligned} \tag{S2}$$

With the application of trigonometric functions, the first component J_1 of Equation (S2) can be transformed into:

$$\begin{aligned}J_1&=\frac{1}{2}(|t_{xx}|e^{i\varphi_{xx}}-|t_{yy}|e^{i\varphi_{yy}})\cdot\begin{bmatrix}\cos2\theta & \sin2\theta\\ \sin2\theta & -\cos2\theta\end{bmatrix}\\
&=\frac{1}{2}(|t_{xx}|e^{i\varphi_{xx}}-|t_{yy}|e^{i\varphi_{yy}})\cdot\{\frac{1}{2}(e^{i2\theta}+e^{-i2\theta})\cdot\begin{bmatrix}1 & 0\\ 0 & -1\end{bmatrix}+\frac{1}{2i}(e^{i2\theta}-e^{-i2\theta})\cdot\begin{bmatrix}0 & 1\\ 1 & 0\end{bmatrix}\}\\
&=\frac{1}{2}(|t_{xx}|e^{i\varphi_{xx}}-|t_{yy}|e^{i\varphi_{yy}})\cdot\{\frac{1}{2}e^{i2\theta}\cdot\begin{bmatrix}1 & -i\\ -i & -1\end{bmatrix}+\frac{1}{2}e^{-i2\theta}\cdot\begin{bmatrix}1 & i\\ i & -1\end{bmatrix}\}\end{aligned} \tag{S3}$$

Then the transmitted electric field can be described as:

$$
\begin{aligned}
\vec{E}_{out} &= (J_1 + J_2)\cdot \vec{E}_{in} \\
&= \frac{1}{2}(|t_{xx}|e^{i\varphi_{xx}} - |t_{yy}|e^{i\varphi_{yy}})\cdot\{\frac{1}{2}e^{i2\theta}\cdot\begin{bmatrix}1 & -i\\ -i & -1\end{bmatrix} + \frac{1}{2}e^{-i2\theta}\cdot\begin{bmatrix}1 & i\\ i & -1\end{bmatrix}\}\cdot\vec{E}_{in} \\
&+\frac{1}{2}(|t_{xx}|e^{i\varphi_{xx}} + |t_{yy}|e^{i\varphi_{yy}})\cdot\vec{E}_{in} \\
&= \frac{1}{2}\ t_{xx} - t_{yy}e^{i\cdot\Delta\varphi}\ [e^{i\cdot 2\theta}\hat{\sigma}_R + e^{-i\cdot 2\theta}\hat{\sigma}_L]\cdot\vec{E}_{in} + \frac{1}{2}\ t_{xx} + t_{yy}e^{i\cdot\Delta\varphi}\ \cdot\vec{E}_{in}
\end{aligned}
\tag{S4}
$$

where it is defined that $\hat{\sigma}_R = \frac{1}{2}\begin{bmatrix}1 & -i\\ -i & -1\end{bmatrix}$ and $\hat{\sigma}_L = \frac{1}{2}\begin{bmatrix}1 & i\\ i & -1\end{bmatrix}$ are the handedness operators, which restrict the polarization state of transmitted wave. Herein, when the polarization state of incident wave is RHCP (or LHCP) as $\vec{E}_{in} = |\vec{R}\rangle = \begin{bmatrix}1\\ -i\end{bmatrix}$ (or $\vec{E}_{in} = |\vec{L}\rangle = \begin{bmatrix}1\\ i\end{bmatrix}$), there exist $\hat{\sigma}_R|\vec{R}\rangle = 0$ (or $\hat{\sigma}_L|\vec{L}\rangle = 0$), and $\hat{\sigma}_L|\vec{R}\rangle = |\vec{L}\rangle$ (or $\hat{\sigma}_R|\vec{L}\rangle = |\vec{R}\rangle$). $\Delta\varphi = |\varphi_{xx} - \varphi_{yy}|$ is the phase difference between the transmission coefficients of the two linear polarizations.

On the other hand, as for circular base, the transmittance properties of position-varied meta-atoms with rotation angle θ can be described by Jones matrix as follow:

$$
\begin{aligned}
\mathbf{T}_{circular}(\theta) &= M(\theta)^T\cdot\begin{bmatrix}\mathbf{t}_{co} & \mathbf{t}_{cross}\\ \mathbf{t}_{cross} & \mathbf{t}_{co}\end{bmatrix}\cdot M(\theta) \\
&= \begin{bmatrix}\cos\theta & -\sin\theta\\ \sin\theta & \cos\theta\end{bmatrix}\cdot\begin{bmatrix}\mathbf{t}_{co} & \mathbf{t}_{cross}\\ \mathbf{t}_{cross} & \mathbf{t}_{co}\end{bmatrix}\cdot\begin{bmatrix}\cos\theta & \sin\theta\\ -\sin\theta & \cos\theta\end{bmatrix} \\
&= \begin{bmatrix}\mathbf{t}_{co}\cdot\cos^2\theta - 2\mathbf{t}_{cross}\cdot\sin\theta\cos\theta + \mathbf{t}_{co}\cdot\sin^2\theta & \mathbf{t}_{cross}\cdot(\cos^2\theta - \sin^2\theta)\\ \mathbf{t}_{cross}\cdot(\cos^2\theta - \sin^2\theta) & \mathbf{t}_{co}\cdot\sin^2\theta - 2\mathbf{t}_{cross}\cdot\sin\theta\cos\theta + \mathbf{t}_{co}\cdot\cos^2\theta\end{bmatrix} \\
&= \begin{bmatrix}\mathbf{t}_{co} - \mathbf{t}_{cross}\cdot\sin 2\theta & \mathbf{t}_{cross}\cdot\cos 2\theta\\ \mathbf{t}_{cross}\cdot\cos 2\theta & \mathbf{t}_{co} + \mathbf{t}_{cross}\cdot\sin 2\theta\end{bmatrix} \\
&= \mathbf{t}_{co}\cdot\begin{bmatrix}1 & 0\\ 0 & 1\end{bmatrix} + \frac{1}{2}\mathbf{t}_{cross}\cdot\{e^{i2\theta}\cdot\begin{bmatrix}i & 1\\ 1 & -i\end{bmatrix} + e^{-i2\theta}\cdot\begin{bmatrix}-i & 1\\ 1 & i\end{bmatrix}\}
\end{aligned}
\tag{S5}
$$

where the subscript of circular transmission coefficients “co” and “cross” represent the relative CP states:

$$
\mathbf{t}_{co} = \mathbf{t}_{LL} = \mathbf{t}_{RR} = \frac{1}{2}[t_{xx}e^{i\varphi_{xx}} + t_{yy}e^{i\varphi_{yy}}] = \cos(\frac{1}{2}\Delta\varphi)\cdot e^{i\cdot\frac{1}{2}\sum\varphi} \tag{6a}
$$

$$
\mathbf{t}_{cross} = \mathbf{t}_{LR} = \mathbf{t}_{RL} = \frac{1}{2}[t_{xx}e^{i\varphi_{xx}} - t_{yy}e^{i\varphi_{yy}}] = \sin(\frac{1}{2}\Delta\varphi)\cdot e^{i\cdot\frac{1}{2}(\sum\varphi-\pi)} \tag{6b}
$$

Where $|t_{xx}| = |t_{yy}| = 1$, and $\sum\varphi = \varphi_{xx} + \varphi_{yy}$ is the summation of propagation phase along fast and slow axes. Herein, $\Phi_1^+ = \frac{1}{2}\sum\varphi$ is defined as original phase, which is provided by propagation phase by varying the dimensions of meta-atoms, and $\varphi'(x, y) = 2\theta(x, y)$ is the additional degree of freedom, which is provided by geometric phase from rotating meta-atoms and only works in the cross-polarized field. It is noted that the response of $\varphi'(x, y)$ under orthogonal CP incidence are conjugate symmetry, attributed to the inherent property of geometric phase.

Text S2. Design principle of meta-atom

Referring to equivalent circuit of filters in microwave region, it is theoretically possible to achieve unity transmittance and full 2π phase coverage by involving high order *LC* resonance in a band-pass filter model, which would provide more degree of freedom for phase tuning.

Here, the basic microwave meta-atom is composed of five metallic layers and four dielectric substrate layers as presented in **Figure S1**(a), realizing a 4th order *LC* band-pass filter. The odd-numbered metallic layers are occupied by rectangular metallic patches, acting as capacitive component in the equivalent band-pass filter circuit. Three parallel gaps are implemented in each rectangular patch for miniaturization purpose. The even-numbered layers constituted by metallic grids with centered circular apertures act as equivalent inductor, guaranteeing the polarization insensitivity during the rotation of the patch layer. The dielectric substrates with similar thicknesses are regarded as transmission lines with impedance Z_n. Each adjacent capacitive and inductive layer can be considered as an *LC* resonant element in the equivalent circuit such that the whole block enables a 4th order resonance to generate a broad operating frequency band with high transmission coefficient and the whole phase coverage of 2π under linearly polarized illuminations.

For the sake of demonstration, one meta-atom is selected to present the EM response in microwave region. The simulated magnitude and phase profiles of transmission spectra under linearly polarized incidence and CP incidence are depicted in Figure S1(b)-S1(c), respectively. It can be observed that under the illumination of *x*- and *y*-polarized incidences, the amplitude of both t_{xx} and t_{yy} is higher than 0.9 and the phase difference $\Delta\varphi = \varphi_{xx} - \varphi_{yy}$ is kept to $\pi/2$ within 9.5 GHz – 10.5 GHz frequency range, guaranteeing the effective generation and conversion of transmitted wave with LHCP and RHCP states. Meanwhile, the amplitude of co-polarized and cross-polarized transmitted coefficient under LHCP incidence can reach 0.7 around the center frequency of 10 GHz as shown in Figures. S1(d)-S1(e), which implies that the transmission coefficient of energy carried by co-polarized and cross-polarized can simultaneously approach 0.5 separately ($t_{co}^2 \approx t_{cross}^2$). The phase difference between co- and cross-polarized coefficients ($\Delta\varphi_{cir} = |\varphi_{co} - \varphi_{cross}|$) is also fixed to $\pi/2$, providing a phase criterion to independently control orthogonal CP output channels in transmitted field. According to the design criterion above, a library of 24 meta-atoms, endowing uniform phase interval of $\pi/12$ in order to obtain the full phase coverage, is established with further parametric optimization. The simulated circularly

polarized phase delays (φ_{co} and φ_{cross}) and corresponding phase difference are exhibited in Figure S1(f), offering the basic effective microwave elements for full manipulation of transmitted wave.

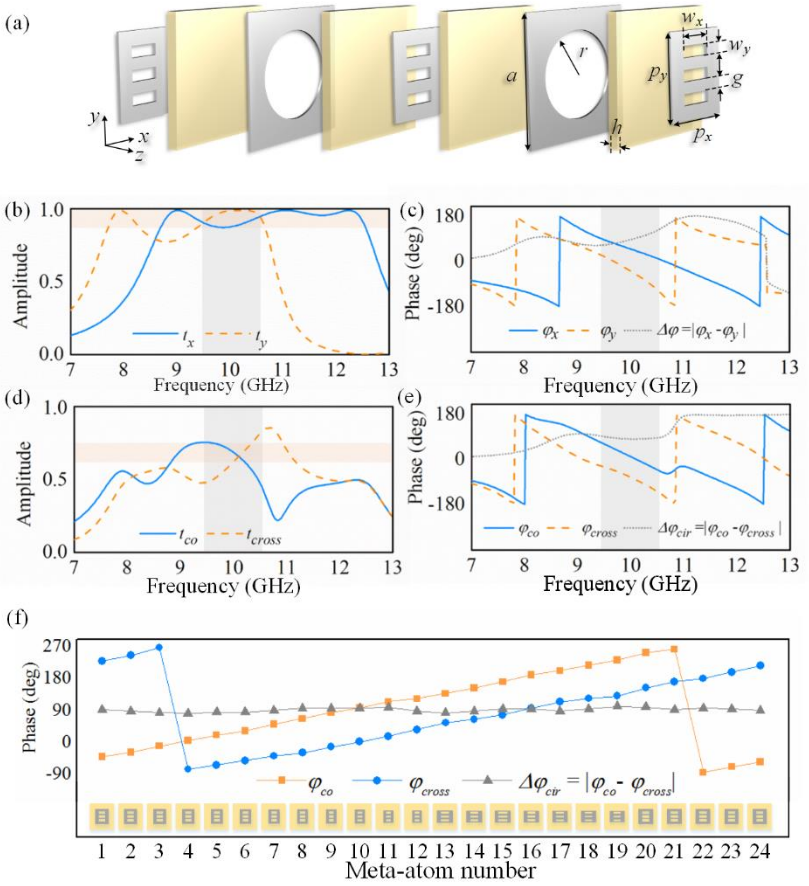

Figure S1. (a) Topological layout of an elementary meta-atom, where periodicity a of meta-atom equals to 8.8 mm, radius of circular aperture in the grid layer is r = 3 mm, thickness of each dielectric substrate is h = 1 mm, p_x and p_y are the length and width of metallic patch, w_x and w_y are the dimensions of rectangular gap in the metallic patch, g is the distance between two adjacent gaps. (b) Simulated amplitude and (c) phase of transmission coefficients of a representative microwave meta-atom under x- and y-linearly polarized incident waves. (d) Simulated amplitude and (e) phase of co-polarized and cross-polarized transmission coefficients under LHCP incident wave. (f) Co-polarized and cross-polarized phase of the 24 meta-atoms are selected and optimized to cover the full 2π phase range with π/12 interval.

Furthermore, some basic electromagnetic characteristics of the proposed meta-atom is illustrated in this part. In microwave region, metal can be regarded as perfect electric conductor and the loss tangent of dielectric materials is usually in the order of 10^{-3}. Thus, the system is supposed as a lossless system in the analyzations and simulations. Since the meta-atom proposed in this paper is symmetric in structure

and totally passive (without any active component), all of the proposed meta-atoms are reciprocal. It means that there exist $S_{12}^{xy} = S_{21}^{yx}$, $S_{12}^{yx} = S_{21}^{xy}$ in the general scattering matrix, where the first (second) subscript presents for the region of incident (scattered) field, and the first (second) superscript shows the polarization state of incident (scattered) wave. Additionally, the reciprocity is verified by simulations as shown in **Figure S2**, where the scattering responses of transmission coefficients are exhibited. Figure S2(a) and S2(b) show the amplitude and phase responses of meta-atom with p_x = 4.8 mm, p_y = 5.7 mm. It can be seen that the transmission curves S_{12}^{xy} and S_{21}^{yx} of the meta-atom are in coincident, while S_{12}^{yx} and S_{21}^{xy} are in same tendency, which successfully verify the reciprocity of proposed structure. For the matching property, theoretical model is supposed to be matched with free space which is convenient for calculating the phase-modulation process. However, since there is inevitable reflection within the design process of meta-atom structures, the practical system would be mismatched. During the construction of metasurfaces, the phase responses of meta-atoms have to approach discrete spatial phase distribution, for example required by deflection or OAM generation. To achieve the phase response as accurate as possible, there would be some optimization process of the meta-atom's phase response and the amplitude response would inevitably be sacrificed. So, equivalent network of the meta-atom is considered mismatched with free space.

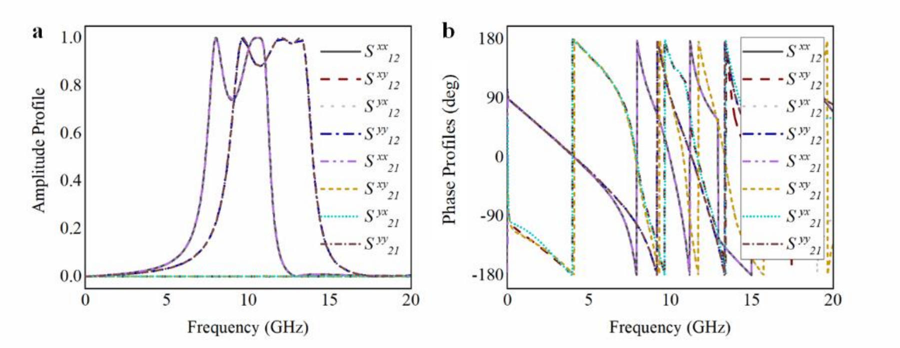

Figure S2 Simulated scattering responses of a representative meta-atoms. (a) amplitude and (b) phase responses.

Text S3. Fabrication process of microwave metadevice sample

The prototypes proposed in this paper are fabricated by using classical printed circuit board (PCB) technique, and which is simply introduced as follows. First, the polyfluortetraethylene dielectric substrates with double copper-cladding layers are prepared. Then the copper surface treatment is conducted for the dry film adhesion purpose, which is used as sensitive material to record printed circuit structures by exposing to ultraviolent (UV) rays. After exposure process, the non-sensitizing dry film needs to be rinsed by sodium carbonate photoresist developer. Furthermore, the exposed copper covered areas are eliminated by specific etching liquid and inner layer etching is performed, and then the process of black oxide for surface roughening and oxidization preventing is completed. On account of the multi-layer samples we proposed, the finally step is the lamination of every treated copper-covered layer and interleaved with adhesive layers.

Based on such PCB technique, the meta-device samples are fabricated and shown in corresponding inset of **Figure S3**, where the insets show zoomed parts for a clear illustration. In our fabrication process, meta-structures are integrated with four pieces of polyfluortetraethylene dielectric-slabs and three adhesive layers. The 1 mm-thick dielectric substrate used has a relative permittivity ε_r = 3.5 and double copper-cladding layer of 0.035 mm-thick, while the adhesive layer has a relative permittivity ε_r = 2.74 and thickness of 0.1 mm. The total thickness of fabricated samples is all 4.475 mm (about $0.15\lambda_0$ at 10 GHz). The proposed metasurfaces all consist of 25 × 25 meta-atoms, which exhibit total size of 220 mm × 220 mm, and the fabrication tolerance is in accordance with initial design requirements.

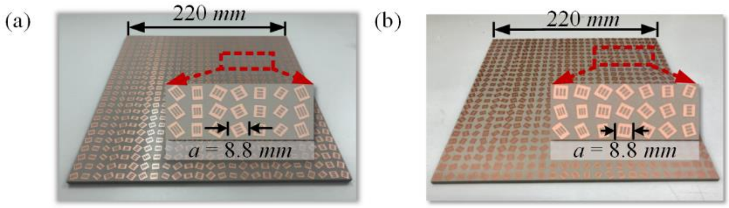

Figure S3. Fabricated samples of (a) metadevice-1 and (b) metadevice-2 with $\Delta\varphi$ = 90°.

Text S4. Measurement in microwave region.

The schematic representation of the near-field measurement system is exhibited in **Figure S4**. Measurements are conducted by a setup surrounded by absorbers in order to minimize parasitic reflections. A 2 GHz - 18 GHz dual-polarized wideband horn antenna is used as the feeding source to launch the circularly polarized quasi-plane wave (LHCP and RHCP), and placed at a distance $d > 20\lambda_0$ away from the meta-devices. A fiber optic active antenna is used as field probe to measure both the amplitude and the phase of the electric field. The probe is fixed on two translation stages controlled by a motion controller and its position is incremented by a step of 2 mm. Both the horn antenna and the field probe are connected to an Agilent 8722ES vector network analyzer, which is adopted to measure the complex S_{11} and S_{21} parameters including both amplitude and phase. Here S_{11} and S_{21} parameters represent the reflection and transmission coefficients of the system under test, respectively. The probe is oriented in two directions in order to measure the two components $\vec{E}_x$ and $\vec{E}_y$ (horizontal and vertical) of the transmitted electric field, and then the CP transmitted field at one fixed pixel can be calculated by $\vec{E}_{co} = \vec{E}_x + i \cdot \vec{E}_y$ for co-pol component and $\vec{E}_{cross} = \vec{E}_x - i \cdot \vec{E}_y$ for cross-polarized component under LHCP incidence, including both amplitude and phase. With the variation of the position of the field probe via the motion controller, the *xoy* and *xoz* planes can be totally covered, and the experimental near-field intensities and phase profiles can be measured.

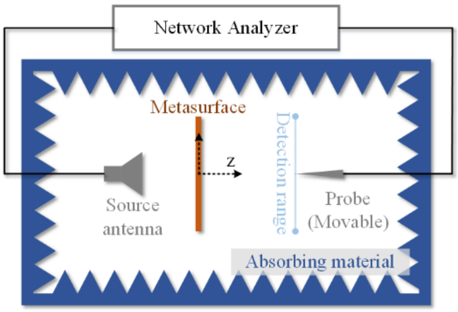

Figure S4. Schematic illustration of the measurement setup for near-field mapping.

Text S5. Scheme demonstration in optical regime

Here, the basic optical meta-atom shown in **Figure S5**(a) is composed of five layers of noble metal film and four silicon dioxide spacers, realizing a 4th-order *LC* band-pass filter as shown in Figure S5(b). A representative meta-atom is selected to present the response of the equivalent broadband filter model. The simulated magnitude and phase profiles of transmission spectra under linearly polarized incidence are depicted in Figure S5(c) and S5(d). It can be observed that under the illumination of *x*- and *y*-polarized light, the amplitudes of both t_{xx} and t_{yy} are higher than 0.8 within 160 THz – 200 THz frequency range, indicating the ultra-broad pass-band performance of proposed equivalent filter model. Meanwhile, the phase difference between *x*- and *y*-polarized transmission coefficients can be kept around π within the working band 160 THz – 200 THz, which can result in the high conversion efficiency of cross-polarized energy. Furthermore, the simulation results of transmission responses, including amplitudes t_{co}, t_{cross} and phase delays φ_{co} and φ_{cross} at 1550 nm varying against the length (p_x) and width (p_y) of the patch are shown in Figure S5(e) under the illumination of LHCP incidence. According to the above variation rule and design criterion, a library of 8 meta-atoms endowing uniform phase interval of π/4 in order to obtain the full 2π-phase range coverage, is established with further parametric optimization. The simulated circularly polarized phase delays φ_{co} and φ_{cross} are displayed in Figure S5(f), offering basic effective elements for full manipulation of transmitted wave.

In order to validate the proposed formalism that can be expanded in optical regime, we conduct a metasurface working with $\lambda_{0\ (\text{opt})}$ = 1550 nm, which can generate converging beam in co-polarized transmitted field and vortex beam carrying OAM with mode l = 1 in cross-polarized transmitted field (same wavefronts with metadevice-1 in main text) under the spin-up illumination. It can be observed from **Figure S6**(a) and S6(b) that, under the impinging of spin-up σ^- illumination, the co-polarized output wavefront is focus beam with focal length $f_{(\text{opt})} = 5\lambda_{0\ (\text{opt})}$, and the vortex beam with OAM l = 1 is generated in the cross-polarized field. When the incident light state changed into spin-down σ^+, the co-polarized output shown in Figure S6(c) performs focusing beam with focal length $f_{(\text{opt})} = 5\lambda_{0\ (\text{opt})}$, which is totally similar to co-polarized output in Figure S6(a). Meanwhile, the cross-polarized output exhibits converged vortex beam with OAM l = -1. The converged vortex energy intensity along propagation direction (focal length is about $1.6\lambda_{0\ (\text{opt})}$) is shown in Figure S6(d), and the corresponding vortex ring-shape energy distribution and helical phase pattern are exhibited in the inset of Figure S6(d). The

optical results indicate that the proposed phase scheme for simultaneous manipulation of both output channels can be effectively expanded into other frequency regime.

Here it should be noticed that the meta-atom is not necessarily to be multi-layered structure. Other metallic or dielectric nano-structures can be appropriate for the fabrication of optical metasurfaces, as long as the two phase-modulation degrees (propagation phase and geometric phase) can be satisfied by tailoring some specific parameters of meta-atom, for example the dielectric pillars shown in 10.1103/PhysRevLett.118.113901.

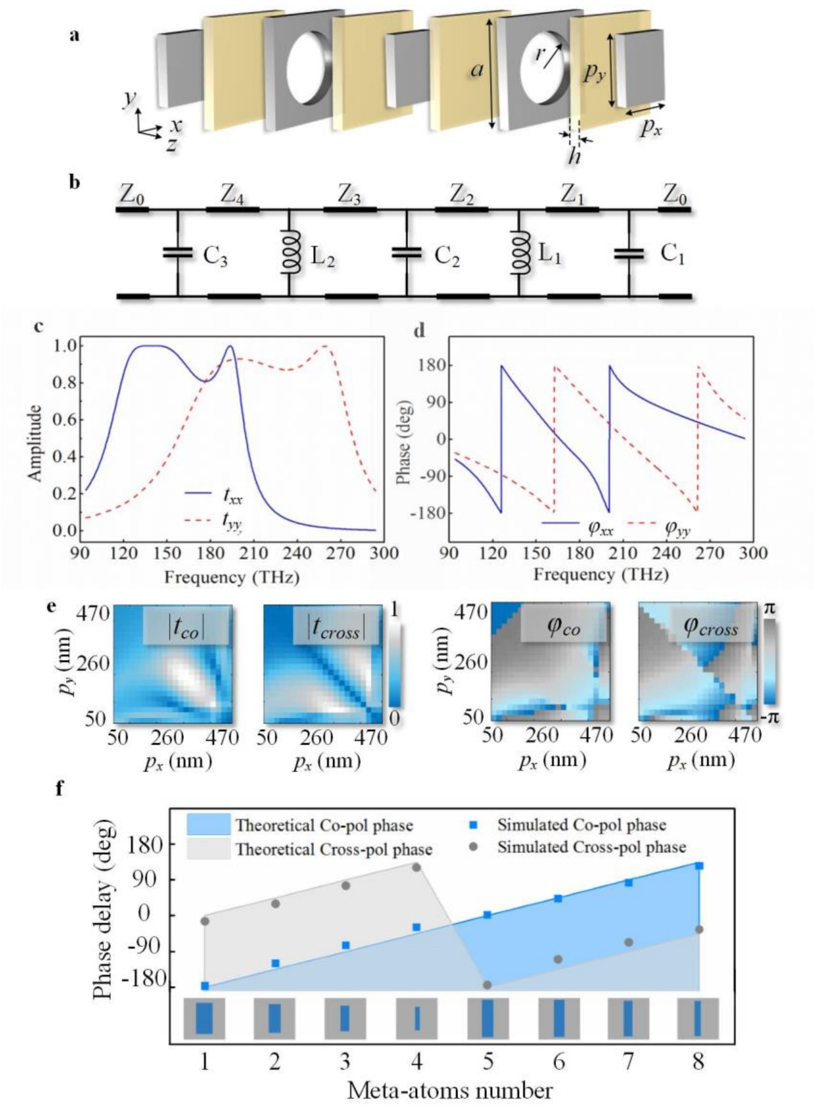

Figure S5. (a) Topological layout of an elementary meta-atom, where periodicity of meta-atom is a = 550 nm, radius of circular aperture in the grid layer is r = 250 nm, thickness of each silicon dioxide dielectric spacer is h = 50 nm, p_x and p_y are the length and width of noble metal patch. (b) Corresponding equivalent 4th-order resonance circuit model of proposed meta-atom. Simulated (c) amplitude and (d) phase spectra of transmission coefficients under the illumination of x- and y- linearly

polarized light. (e) Simulated amplitude and phase spectra under spin up incidence at 1550 nm when the length and width of patch layer are varied from 50 nm to 470 nm with 20 nm sampling step. (f) Co-polarized and cross-polarized phase of the 8 meta-atoms selected and optimized to cover the full 2π phase range with π/4 interval.

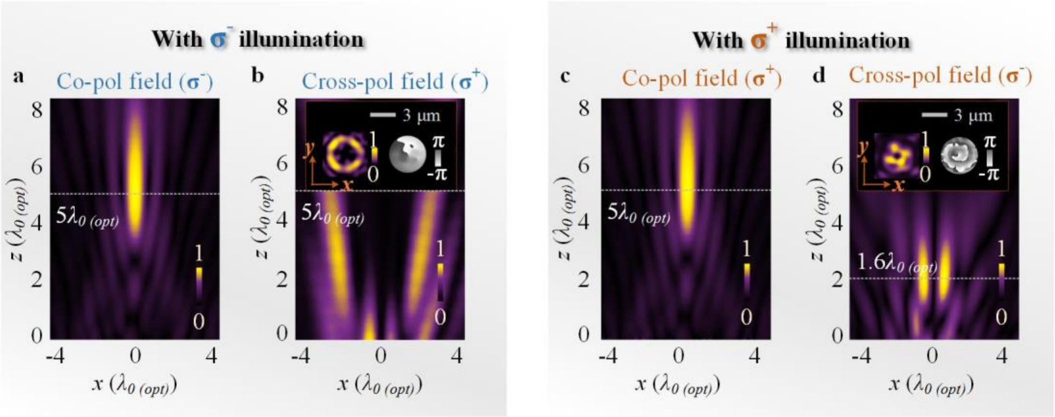

Figure S6. Demonstration of optical metadevice for independent manipulation of co- and cross-polarized output wavefronts. With the spin-up illumination, (a) the co-polarized focusing beam output and (b) cross-polarized vortex beam carrying OAM mode l = 1, the insets show the energy and phase distributions in *xoy* plane with $z = 5\lambda_0$. With the spin-down illumination, (c) the co-polarized focusing beam output and (d) cross-polarized converged vortex beam carrying OAM mode l = -1, the insets show the energy and phase distributions of vortex beam in *xoy* plane with $z = 1.6\lambda_0$.

Text S6. Evaluation of efficiency of the proposed metadevices

The efficiency of meta-device is evaluated and discussed in this part, and two efficiency characteristics are defined here, including wavefront utilization efficiency (UE) and transmission efficiency (TE). The energy of the required field can be obtained by the sum of measured intensity at each point in the detecting plane, which is collected from the probe and analyzed in the vector network analyzer as detailed in Text S4. The incident energy is obtained by the same process but without placing the fabricated sample. Different from the measured energy intensity, which is obtained by graphing the energy values point-by-point in the detection plane, the efficiency against frequency is acquired by integrating all the energy values on the whole detection plane. Here, the transmission efficiency can be calculated by:

$$TE = \frac{P_{out}}{P_{in}} = \frac{\int \left|\vec{E}_{out}\right|^2 ds}{\int \left|\vec{E}_{in}\right|^2 ds} \qquad \text{(S7)}$$

where P_{out} and E_{out} expresses the total transmitted energy and electric field, P_{in} and E_{in} express the incident energy and electric field, and s is the area of the measured plane. The measured TE of fabricated metadevice-1 and metadevice-2 with $\Delta\varphi$ = 90° are 77% and 82% at the center frequency 10 GHz, respectively. These results prove the transmission abilities of the designed metadevices and guarantee the high performances of the required functionalities.

Furthermore, the utilization efficiency can be analyzed by:

$$UE = \frac{P_{cir}}{P_{out}} = \frac{\int \left|\overrightarrow{E_{co}}\right|^2 ds + \left|\overrightarrow{E_{cross}}\right|^2 ds}{\left|\overrightarrow{E_{out}}\right|^2 ds} \qquad \text{(S8)}$$

where P_{cir} is the total energy with circular polarizations (both LHCP and RHCP) in the transmitted field, representing the sum of both co-polarized and cross-polarized components. It can be seen in inset of Figure 4h in the main text that the wavefront utilization efficiency for all the metadevices are approaching 100% within the measured frequency range of 9 GHz - 11 GHz, which indicates that almost all energy in the transmission can be modulated to perform corresponding preset functionalities. Only a little part of energy is lost at several frequency points due to the uncompleted CP conversion of several meta-atoms. Indeed, some meta-atoms do not achieve the exactly required $\Delta\varphi$ between $\vec{E}_x$ and $\vec{E}_y$, resulting in the generation of undesired elliptically polarized transmitted components.
